# Utilization of Wearable Devices as means for Remote Digital Biometric Data Collection in a community‐based, rural randomized controlled trial among Alzheimer’s disease dyads

**DOI:** 10.1002/alz.090883

**Published:** 2025-01-09

**Authors:** Elizabeth K. Rhodus, Md Saif Hassan Onim, Celeste Roberts, Sanjeev Kumar, Amer M. Burhan, Himanshu Thapliyal

**Affiliations:** ^1^ University of Kentucky, Lexington, KY USA; ^2^ University of Tennessee, Knoxville, TN USA; ^3^ Temerty Faculty of Medicine, University of Toronto, Toronto, ON Canada; ^4^ Ontario Shores Centre for Mental Health Sciences, Whitby, ON Canada

## Abstract

**Background:**

Use of remote measurement of physiological parameters using digital biometrics (i.e., Electro Dermal Activities, heart rate, oxygen saturation, blood volume pulse, etc.) has a multitude of opportunities in rural contexts that have not yet fully been explored in Alzheimer’s disease (AD) clinical research. This study assessed feasibility and acceptability of advanced digital biometric data collection among rural, community‐dwelling adults living with Alzheimer’s disease with support from primary caregivers (dyad).

**Methods:**

Initial feasibility and acceptability of a wrist‐worn wearable device (Empatica E4) as means for digital biometric data collection were assessed among participants of a larger randomized controlled trial which was aimed to assess a non‐pharmacological care intervention improving behavioral symptoms of AD. Following consent, dyads were mailed an E4 device and behavior tracking journal to companion digital recordings for one week of data collection. Caregivers assisted the person with AD in wear and care of the E4 device and completed time‐stamped behavior tracking. Feasibility was assessed based on completeness of digital biometric data recordings and acceptability was determined based on wear schedules for the device in participants with AD (worn >50% of allotted time).

**Results:**

Digital biometric data collection via remote, wearable devices is feasible and acceptable among participants with AD in rural settings among 16 dyads (person with AD age x̄=77±2.4 years, 10 female; caregiver age years x̄=57±2.9 years, 8 female). The E4 correctly captured biometric signals (Electro Dermal Activities, blood volume pulse, Skin Temperature, etc.). The device was acceptable as 14 of the 16 participants with AD wore the device >50% of allotted time with proper completion of the caregiver‐reported behavior tracking. Further, we observed that unsupervised machine learning models were able to create digital biometrics that mirrored caregivers’ notes.

**Conclusion:**

Advances in technology, evolution of health status surveillance, and vast needs in rural areas create an ideal scenario to advance research in digital biometrics. With goals to use digital biometrics as primary outcome measures in AD clinical trials, the findings presented here illustrate feasibility and acceptability among geographically remote communities. Additional research is needed to assess context‐aware machine learning applicability in intervention‐based clinical research.

